# Rationale and design of SuPPoRT: a multi-centre randomised controlled trial to compare three treatments: cervical cerclage, cervical pessary and vaginal progesterone, for the prevention of preterm birth in women who develop a short cervix

**DOI:** 10.1186/s12884-016-1148-9

**Published:** 2016-11-21

**Authors:** Natasha L. Hezelgrave, Helena A. Watson, Alexandra Ridout, Falak Diab, Paul T Seed, Evonne Chin-Smith, Rachel M. Tribe, Andrew H. Shennan

**Affiliations:** Division of Women’s Health, Faculty of Life Sciences & Medicine, King’s College London, Women’s Health Academic Centre King’s Health Partners, 10th Floor North Wing, St Thomas’ Hospital Campus, London, SE1 7EH UK

**Keywords:** Preterm birth, Arabin pessary, Cerclage, Progesterone, Cervical length, Biomarkers

## Abstract

**Background:**

Clinically, once a woman has been identified as being at risk of spontaneous preterm birth (sPTB) due to a short cervical length, a decision regarding prophylactic treatment must be made. Three interventions have the potential to improve outcomes: cervical cerclage (stitch), vaginal progesterone and cervical pessary. Each has been shown to have similar benefit in reduction of sPTB, but there have been no randomised control trials (RCTs) to compare them.

**Methods:**

This open label multi-centre UK RCT trial, will evaluate whether the three interventions are equally efficacious to prevent premature birth in women who develop a short cervix (<25 mm on transvaginal ultrasound). Participants will be asymptomatic and between 14^+0^ and 23^+6^ weeks’ gestation in singleton pregnancies. Eligible women will be randomised to cervical cerclage, Arabin pessary or vaginal progesterone (200 mg once daily) (*n* = 170 women per group).

The obstetric endpoints are premature birth rate <37 weeks’ of gestation (primary), 34 weeks and 30 weeks (secondary outcomes) and short-term neonatal outcomes (a composite of death and major morbidity). It will also explore whether intervention success can be predicted by pre-intervention biomarker status.

**Discussion:**

Preterm birth is the leading cause of perinatal morbidity and mortality and a short cervix is a useful way of identifying those most at risk. However, best management of these women has presented a clinical conundrum for decades.

Given the promise offered by cerclage, Arabin pessary and vaginal progesterone for prevention of preterm birth in individual trials, direct comparison of these prophylactic interventions is now essential to establish whether one treatment is superior. If, as we hypothesise, the three interventions are equally efficacious, this study will empower women to make a choice of treatments based on personal preference and quality of life issues also explored by the study.

Our exploratory analysis into whether the response to intervention is related to the pre-intervention biomarker status further our understanding of the pathophysiology of spontaneous preterm birth and help focus future research questions.

**Trial registration:**

EudraCT Number: 2015-000456-15. Registered 11th March 2015

## Background, including rationale and previous systematic reviews

There are 12.9 million premature births annually worldwide [1] and despite the magnitude of the problem, there is no established early pregnancy screening test or effective treatment for women once a high risk of spontaneous preterm birth (sPTB) is ascertained. The associated morbidity, mortality and high health costs are well documented. Only 39% of those infants born <26 weeks’ gestation survive and 13% of these suffer severe cerebral palsy or sensory impairment [2]. Despite considerable efforts to introduce new therapies for the prevention and treatment of spontaneous preterm labour, sPTB rates are still rising; 6% of births in the UK are premature. sPTB is thought to be the result of multiple aetiologies influenced by a wide number of genetic, biological, psychosocial and environmental factors (e.g. multiple pregnancy, infection, placental abruption and stress) yet the chronology and aetiology of sPTB is insufficiently understood. Early sPTB likely results from a complex interaction of maternal and/or fetal inflammatory responses that culminate in progressive cervical shortening and myometrial contractions.

Detection of a short cervix using transvaginal ultrasonography has emerged as a useful predictor of sPTB in both low and high risk pregnancies [3, 4] and risk of preterm birth is inversely related to cervical length; the shorter the cervix, the higher the risk of preterm birth. In clinical practice, once a woman has been identified as being at higher risk of sPTB by virtue of a short cervix, a decision regarding prophylactic treatment must be made. Three interventions have been proposed to treat patients with a short cervix; cervical cerclage [5, 6], cervical pessary [7] or vaginal progesterone therapy [8–10]. There is little evidence to guide clinicians as to which of these three interventions is the best to use and so the treatment received predominantly depends on the centre a woman is treated in and her clinician’s preference.

A cerclage is the insertion of a ‘purse string’ suture around the cervix under regional anaesthesia. There is little consensus on the optimal procedure or technique (e.g. low/high vaginal, abdominal, tape/nylon, single/multiple, endocervical/purse string) or timing of insertion (elective, ultrasound indicated, pre-conceptual). Furthermore, the mechanism of action is not understood; cerclage may offer a degree of structural support, but also plays a role in maintaining a biochemical barrier protecting membranes against exposure to ascending pathogens. It also is known to induce an inflammatory response, which may encourage tissue repair. A ‘history indicated’ cerclage is inserted in early pregnancy (8–14 weeks’) in women who have a history of late miscarriage or preterm birth. The largest randomised controlled trial comparing history-indicated cerclage with expectant management (*n* = 1292), demonstrated that benefit of cerclage was only seen in women with three prior fetal losses/premature deliveries, where their risk of preterm birth reduced by more than half [6].

‘The benefit of cerclage following confirmation of cervical shortening (ultrasound indicated cerclage), has been reported in a subgroup of high risk women (history of preterm second-trimester loss or birth before 36 weeks of gestation) who have a cervix <25 mm in length, with meta-analysis [5] demonstrating a significant reduction in delivery before 35 weeks of gestation [relative risk (RR) 0.57; 95% confidence interval (CI) 0.33–0.99] for women with prior second trimester miscarriage, and RR 0.61; 95% CI 0.40–0.92 for women with prior preterm birth] when compared with expectant management.’

The Arabin pessary is a round and cone shaped flexible silicon device which is designed to be inserted into the vagina and sit in the upper fornix, to support and incline the cervix, with the intention to prevent premature cervical shortening and preterm birth. Goya et al. [7], [11] reported a multicentre randomised controlled trial (*n* = 385) on pessary use in unselected women screened by transvaginal ultrasound (TVS) and showed that in women with a short cervical length (<25 mm) between 18 and 22 weeks, the pessary reduced the rate of sPTB <34 weeks’ gestation compared with controls (6% *vs* 27%, odds ratio 0 · 18, 95% CI 0 · 08 to 0 · 37; *p* < 0 · 0001), with a significant difference detected in the occurrence of composite poor neonatal outcome. In a subsequent smaller RCT, 108 Asian women with a singleton pregnancy and a cervical length <25 mm at routine second-trimester TVS were randomized to either pessary or control group. The mean gestational age at delivery was 38.1 weeks in the pessary group compared with 37.8 weeks in the expectant management group, with no significant differences in the rates of delivery before 28, 34 or 37 weeks [12].

Vaginal progesterone is given as a potential therapy to sPTB in singleton pregnancies based on a Cochrane systematic review [8] reporting that prophylactic progesterone (intramuscular and vaginal administration of varying doses) was associated with a significant reduction in preterm birth <34 weeks (one study; 142 women; RR 0.15; 95% CI 0.04–0.64) and preterm birth at less than 37 weeks (four studies; 1255 women; RR 0.80; 95% CI 0.70–0.92). A meta-analysis of vaginal progesterone treatment given to all women with a short cervix <25 mm showed a reduction in preterm birth at <33 weeks; [RR, 0.58; 95% confidence interval [CI], 0.42 to 0.80), <35 weeks (RR, 0.69; 95% CI, 0.55 to 0.88), and <28 weeks (RR, 0.50; 95% CI, 0.30 to 0.81) and neonatal composite morbidity and mortality (RR 0.57; 95% CI, 0.40 – 0.81) [9]. A recent large study suggested a lack of efficacy of vaginal progesterone and no long-term benefit at two years, adding to the uncertainty around progesterone use [10].

The plasma (and/or uterine) concentration of progesterone required to reduce sPTB is unknown, and the mechanism of action uncertain. It is, however, frequently used in clinical practice (usual dose 200–400 mg). The doses of vaginal progesterone used in completed trials are 100 mg (*n* = 142) or 200 mg (*n* = 250) [13]. Whilst vaginal progesterone use for the prevention of sPTB is unlicensed, it is commonly used in clinical practice in the UK and Europe and is also recommended for use in the USA by the Society for Maternal and Fetal Medicine. NICE recommends vaginal progesterone in women between 16^+0^ and 24^+0^ weeks with a cervical length <25 mm and no history of late miscarriage or sPTB.

Progesterone or cerclage are advised by NICE for women with a history of late miscarriage or preterm birth [14]. Currently cervical cerclage is the Royal College of Obstetricians (RCOG) recommended treatment for women with a short cervix <25 mm and a history of 1 or more late miscarriages or preterm birth [15].

However, there have been no direct comparison of these three interventions to inform guidelines as to the optimal management of all high risk women who develop a sonographic short cervix. Women at high risk of sPTB with a short cervix cannot, at present, be counselled as to which is the most suitable intervention to reduce their risk of delivering prematurely.

Our research group recently published an exploratory observational study [16] to evaluate the relationship between pro-inflammatory cytokines, cervical shortening and intervention, within which 37 women who developed a short cervix were randomised to treatment with vaginal progesterone (*n* = 17) or cervical cerclage (*n* = 19). A clinically important trend towards benefit (gestation at delivery) was noted in the cerclage group (mean gestation 33.7 weeks’ cerclage versus 31.5 weeks’ progesterone) although this result did not achieve statistical significance.

Alfirevic et al., [17] retrospectively compared three cohorts of women with previous sPTB <34 weeks and short cervix treated with cerclage (*n* = 142), vaginal progesterone (*n* = 59) or a pessary (*n* = 42). There were no significant differences in rates of perinatal loss, neonatal morbidity or sPTB, apart from a higher rate of sPTB before 34 weeks’ gestation in the vaginal progesterone *vs* pessary groups. It was concluded that randomized comparisons of these three management strategies, or combinations thereof, are needed to determine optimal management. If a randomised study, such as the one described here, showed that the three treatments were equally efficacious, then women and clinicians would have greater choice regarding treatment plans, expensive surgery (and potential complications) could be avoided. The pessary could be inserted at a later gestational age, when cerclage is no longer performed, potentially in an outpatient setting.

This randomised controlled trial will answer the current clinical dilemma of which is the most effective method to treat women at high risk of sPTB who develop a short cervix.

### Hypothesis

Current treatments for ultrasound indicated cervical shortening in women at risk of preterm birth confer equal benefit in terms of reducing the numbers of births <37 weeks of gestation.

### Aim

In a randomised controlled trial, to compare three evidence-based treatments for a short cervix detected by ultrasound scan in women at high risk of premature birth: cervical cerclage, cervical pessary and vaginal progesterone therapy.

## Objectives

### Primary objectives

1. To determine if treatment with cervical cerclage, cervical pessary or vaginal progesterone in women at high risk of preterm birth who develop a short cervix by ultrasound measurement are equally efficacious to improve obstetric outcome by lengthening pregnancy and reducing the incidence of preterm delivery before 34 weeks’ gestation.
2. To evaluate the impact of the interventions on short-term neonatal outcomes, assessed as a composite of perinatal death (within 28 days) and major morbidity.

### Secondary objectives

3. To undertake an exploratory analysis to determine whether the response to intervention for a short cervix is related to the pre-intervention inflammatory biomarker status (cervicovaginal fluid (CVF), blood).
4. To evaluate the acceptability to women and clinicians of each of the three treatment arms.
5. To assess the impact of management strategies on health economic outcomes for mother and infant in terms of number of nights in each hospital setting; cost data to hospital discharge/28 days postnatal (it is anticipated that a 6 month and 2 year follow up may be performed if funding is obtained).

### Centres

As a National Institute for Health Research registered portfolio study, SuPPoRT is open to UK hospitals with the appropriate facilities and experience of preterm birth surveillance and treatment.

### Design

**Fig. 1:**
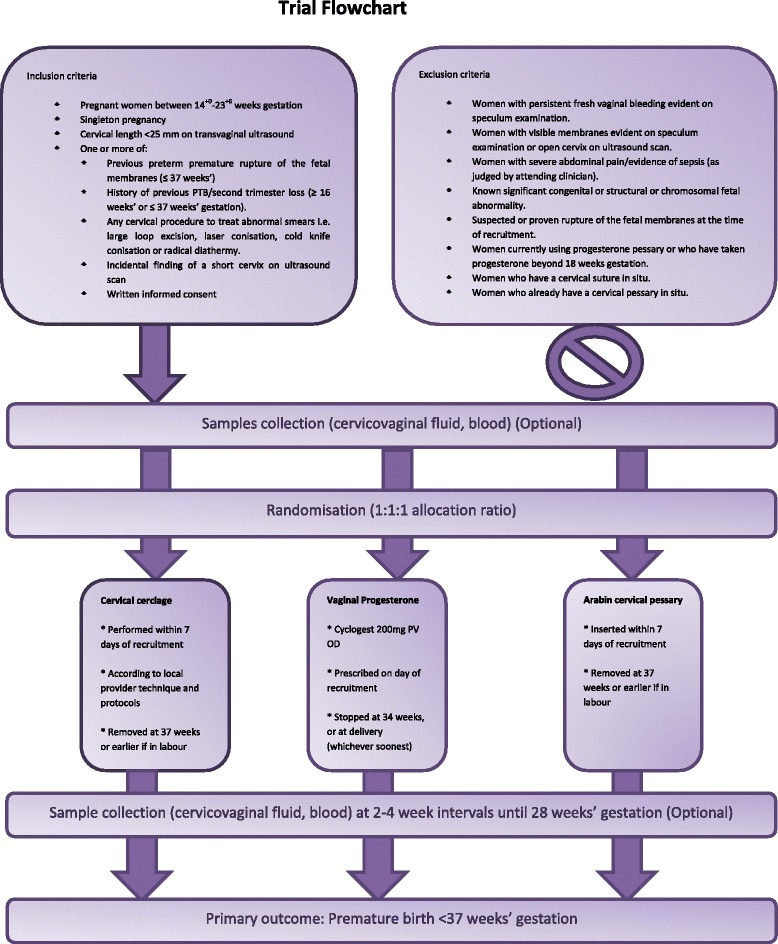
Flow chart of participants in the trial

An open label, multi-centre three armed randomised controlled trial, with an embedded biomarker study, to explore three treatments (cerclage, cervical pessary and vaginal progesterone) for a short cervix in pregnancy (Figure 1).

Women who develop a short cervix will be randomised to one of 3 treatments: cervical cerclage (procedure to take place within 7 days of diagnosis, removed at 37 weeks’), cervical pessary (inserted at diagnosis and removed at 37 weeks’), vaginal progesterone (200 mg once daily per vagina until 34 weeks’ gestation from time of randomisation). At an appropriate time-point between time of randomisation and time of intervention, women will provide a CVF sample and blood sample (for biomarker analysis, optional, if facilities allow). These will be repeated every approximately every two weeks according to routine clinic visits thereafter until 28 weeks’ gestation.

## Eligibility and exclusion criteria

### Eligibility criteria

High risk women with singleton pregnancies who are found to have cervical length <25 mm on transvaginal ultrasound between 14^+0^ weeks’ gestation (dated by ultrasound or last menstrual period and adjusted for ultrasound estimated date of delivery once ultrasound performed if no miscarriage prior to dating ultrasound) until 23^+6^ weeks’ gestation with written consent to participate and one or more of the following risk factors;

- History of

-   o Previous preterm premature rupture of the fetal membranes (≤37 weeks’)
-   o History of previous sPTB/second trimester loss (≥16 weeks’ or ≤ 37 weeks’ gestation).
-   o Any cervical procedure to treat abnormal smears i.e. large loop excision, laser conisation, cold knife conisation or radical diathermy.

Women with an incidental finding of a short cervix on ultrasound scan (e.g. at the time of anomaly scan) are also eligible for inclusion in the study.

### Exclusion criteria

- Women with persistent fresh vaginal bleeding evident on speculum examination.
- Women with visible membranes evident on speculum examination or open cervix on ultrasound scan.
- Women with severe abdominal pain/evidence of sepsis (as judged by attending clinician).
- Known significant congenital or structural or chromosomal fetal abnormality.
- Suspected or proven rupture of the fetal membranes at the time of recruitment.
- Women currently using progesterone pessaries or who have taken progesterone beyond 18 weeks gestation.
- Women who have a cervical suture in situ (vaginal or abdominal).
- Women who already have a cervical pessary in situ.
- If the attending clinician feels that an individual woman is more suited to one treatment modality over another.
- Insufficient understanding of the trial in the opinion of the Investigator.
- Any contraindications or cautions to the investigational medicinal product including:
  known allergy or hypersensitivity to progesterone, hepatic dysfunction, undiagnosed vaginal bleeding, mammary or genital tract carcinoma, thrombophlebitis, thromboembolic disorders, cerebral haemorrhage or porphyria.

### Concomitant medication

Participants will be permitted to use any concomitant medication (aside from the other study products themselves) required alongside the study drug/procedure/device. They will be enquired about and recorded at every visit. Any other medication or treatment that would form normal clinical management for these women at risk of preterm labour, i.e. antibiotics, corticosteroids, tocolytics, etc. will be permitted according to local hospital guidelines and clinician preference.

## Methods

### Recruitment: Identification and consent of participants

Women will be recruited from hospital centres that perform transvaginal ultrasound cervical length measurement routinely for women at high risk of preterm birth. High risk pregnant women who are found to have a cervical length of <25 mm (lowest of 3 measurements) during attendance at high risk surveillance antenatal clinic (hereafter referred to as prematurity surveillance clinic) will be identified. Their case notes will be reviewed for the patient’s potential recruitment into the trial and eligible patients will be informed of the study at time of diagnosis of short cervix. Members of the research team (midwives, doctors and scanning practitioners) will be familiar with the study so can discuss the research with women when required.

All participants will be provided with a written patient information leaflet with verbal translation available for non-English speaking women (via Language Line where available). Women will be consented by an appropriately trained (Good Clinical Practice) doctor. Women will be given information about the study and will be allowed adequate time (up to 48 hours, depending on length of cervix and urgency of treatment, as determined by the attending clinician) to read the patient information sheet and provide written informed consent. If a participant does not consent to sample collection (CVF, saliva, blood), or if the study site does not have the facilities to collect and process samples, this does not preclude trial entry.

### Randomisation

Once written informed consent has been given, the participant will be randomly assigned (1:1:1) to cerclage, progesterone or pessary. Randomisation will be carried out online via the Medscinet web portal (www.medscinet.net). Users will be assigned a personal identifier number. Due to the nature of the interventions, the study is not blinded to the clinician or patient but recruiters and trial coordinators will not have access to the randomisation sequence. Women will be informed at time of recruitment to which arm they have been randomised. A ‘minimisation’ procedure, using a computer-based algorithm, will be used to avoid chance imbalances in important stratification variables. Stratification variables will be a) gestation, b) BMI <30 or >30 kg/m^2^) risk factor (previous premature delivery <24 weeks & previous cervical surgery). Medscinet will write the randomisation program and hold the allocation code. Contact information will be obtained from the patient. Demographic measures will be entered into the central trial database. Following randomisation, the obstetrician will then arrange for the intervention to be performed as the randomisation indicates. There is no ‘emergency code break’ procedure as the trial is an open label RCT.

### Intervention (14 + 0-23 + 6 weeks’ gestation)

#### Cervical cerclage

The cerclage procedure will be booked at the time of randomisation. It will be performed (according to local practice and procedures) within 7 days of recruitment to the trial. A vaginal cerclage will be inserted in the operating theatre by a clinician trained in the procedure, according to the technique preferred by the clinician. It is usually inserted under regional anaesthetic. Tocolysis, antibiotics and antenatal corticosteroids can be considered at the clinician’s preference, but will be documented and considered in the analysis. The patient will usually go home the same day. The suture can be removed easily by exposing the cervix and cutting the knot, usually without the need for anaesthetic. This will be done electively when a woman is 37 weeks’ gestation, or if she presents in symptomatic preterm labour before labour becomes established, to avoid cervical trauma. The suture will also be removed if there is clinical evidence of chorioamnionitis.

#### Vaginal progesterone

Vaginal progesterone (200 mg once daily) will be prescribed at the time of randomisation. Patients will be shown how to insert one progesterone pessary every day until 34 weeks’ gestation (or delivery, whichever is soonest). If the cervix shortens and membranes are visible, prior to 24 weeks’ gestation, a rescue cerclage will be inserted, according to local protocols.

### Dose changes

No dosage adjustments are permitted. Women recruited will be at high risk of early delivery and therefore likely to be highly motivated to comply with treatment. We will monitor compliance carefully by asking local staff to review medication packs. Patients will be asked to return any unused medication at a 34 weeks’ visit (or after delivery) whichever is soonest, and this will be recorded in the online database. If progesterone has been stopped for reasons other than those listed above it can be restarted at any time up to 34 weeks of gestation.

#### Cervical pessary

The appropriately sized pessary will be inserted within 7 days of randomisation by the attending clinician, who will be trained in the procedure. They will be given detailed written instructions about its subsequent management. It will be removed by a trained clinician at 37 weeks’ gestation (or in the event of established labour). If the cervix shortens and membranes become visible prior to 24 weeks’ gestation, a rescue cerclage will be inserted, according to local protocols.

### Study assessments

A summary of study visits is given in Table 1.

**Table 1:** Summary of study visits

Procedure	Screening & randomisation 14^+0^-23^+6^ weeks gestation	Intervention (day 0) 14^+0^-23^+6^ weeks gestation	Baseline visit (day) 1–4 weeks after procedure	Follow up visits (if clinically indicated)	Final visit 34^+0^-38^+6^ weeks’ gestation	Note review (no visit required)
Visit window (± days)	−7 to 0 days	Day 0	7-28 days	Approximately 2–4 weekly	3	Discharge from Hospital or 28 days postnatal
Informed consent	✓					
Medical history and concomitant medications	✓		✓	Optional	✓	
Transvaginal ultrasound Scan	✓		✓	Optional		
Biological sample collection	Optional		Optional	Optional		
Adverse events (AEs) changes to interventions			✓	As needed	✓	✓
Count of un-used medication					✓	
Pessary/Cerclage removal					✓	

### Pre-intervention biomarker measurement

Women will provide biological samples at a convenient time between randomisation and intervention in order to obtain biomarker levels (if study site facilities allow and women consent). This will include one sterile speculum examination and approximately 4 cervico-vaginal swabs (high vaginal and/or endocervical), a swab to measure quantitative fetal fibronectin (qfFN) will be performed from 18 weeks’ gestation, and two 15 ml blood samples, one for genetic analysis; one for biomarker analysis. These samples will be repeated at each subsequent routine clinic visit until 28 weeks’ gestation. Sample collection is not mandatory for trial participation. Some women may only want to provide samples at a single visit or only provide one or more of the samples listed.

### Subsequent study visits

All participants will be seen in a follow up appointment, normally between 2 and 4 weeks post-intervention. Subsequently, all follow up visits will be at the discretion of the attending clinician. Each visit will be documented on the online database. A TVS cervical length measurement should be performed, and results recorded on the trial database, at each visit. At each visit, information will be obtained on compliance, adverse events or pregnancy complications.

The final study visit will take place between 34^+0^ and 38^+0^ weeks’ gestation. Patients randomised to progesterone will be asked to return all unused medication and empty blister packs, in order to collect compliance information. Patients randomised to pessary will return at 37 weeks for removal. Patients randomised to cerclage will be seen between 34 and 37 weeks, and an appointment made for removal in the appropriate clinical area. If delivery has occurred prior to this time, then study staff will contact the participants, to arrange a follow up visit and collection of unused medication. Other than the baseline post intervention visit and final study visit, all other trial visits will be timed to routine clinic attendances according to local clinical protocols and clinician practice. Interim study data will therefore only be collected if the patient attends the appropriate department for routine purposes. Note review will take place 28 days after delivery to capture postnatal maternal, fetal and health economic data.

### Linkage of data

At the time of recruitment a unique study number will be allocated to the patient. Data will be recorded on a password protected KCL computer in order that recruits can be contacted and delivery outcomes recorded. The anonymised research record will not contain any patient identifiable data. All records will be anonymised at time of data entry in accordance with the Data Protection Act 1998. Women will be followed up until postnatal discharge. Paper copies of consent forms will be stored separately and numerically (by study ID) and kept in a secure location in accordance to the Data Protection act 1998.

### Withdrawal from study

Participation in the study is voluntary. A patient has the right to discontinue drug/pessary or completely withdraw from the study at any time, for any reason. Identifiable data or tissue already collected with consent will be retained and used in the study with the women’s permission. Consent would be sought to collect participant’s delivery details (if delivery has not yet occurred). If the participant is withdrawn due to a serious adverse event, the Principal Investigator will arrange for follow-up visits or telephone calls until the event has resolved or stabilised. However as the participants are pregnant women outcome data will be collected routinely (i.e. until delivery) and used in the analysis unless the consent is specifically refused by the participant.

### Expected duration of the trial

The end of the trial will be defined as 28 days post-delivery or discharge from hospital (whichever sooner) of the last recruited participant and infant. At least three centres will be involved, each receiving referrals from satellite units, and will recruit over a 36 month period.

## Outcomes

### Primary Endpoint

Delivery < 37 completed weeks’ gestation (powered).

### Secondary endpoints

- Adverse perinatal outcome, defined as a composite outcome of death (antepartum/intrapartum stillbirths plus neonatal deaths prior to discharge from neonatal services) or one (or more) of intraventricular haemorrhage, periventricular leukomalacia, hypoxic ischemic encephalopathy, necrotizing enterocolitis, bronchopulmonary dysplasia and sepsis.
- Delivery <30 & 34 completed weeks’ gestation.
- Gestation at delivery.
- Time between intervention and delivery.
- Requirement for rescue cerclage (bulging fetal membranes).
- Other maternal and fetal outcomes: clinical course, therapies administered, maternal and fetal morbidity and mortality data until discharge or 28 days postnatal (whichever soonest), adverse effects related to intervention.
- Participant and clinician’s perception of treatment/satisfaction: questionnaires with a selection of participants at 0–2 weeks post procedure. Questionnaires at one year are planned if funding is obtained.
- Health costs at 28 days postnatal.
- Biochemical end-points (on available samples): cervicovaginal swabs will be taken to determine the presence of infection and concentrations of biomarkers of preterm birth, infection and inflammation, and blood samples taken for inflammatory markers and genetic analysis. Results will be correlated with maternal and fetal outcomes.

### Statistical analysis plan including sample size and power calculations

Our previous experience (captured by a robust database of outcome data from >2000 women attending our prematurity clinic) indicates that approximately 50% of women with short cervices (<25 mm) treated with cerclage) deliver early (<37 weeks). From existing published evidence, we have good reason to believe that cerclage, vaginal progesterone and silicone Arabin pessary are all of approximately equal efficacy and reduce the rate of prematurity in women from 75% (untreated) to around 50% [6, 7, 9, 17]. We therefore determine to confirm this by a 3-arm equivalence study. Equivalence is defined as agreement to within 20% (e.g. 40% to 60%). In accordance with Jones et al. [18], we allow for differences in both directions in calculating the power. Complete data on 170 women per arm (510 in all) will give us 90% power to detect clinically important differences of 20% or more in either direction. To allow for dropouts, we aim to recruit 540 women in total.

Analysis will be according to intention to treat. The main outcome is delivery before 37 weeks'. Results will be presented as both odds ratios and risk differences, leading to number needed to treat (NNT) if appropriate, according to CONSORT guidelines. Given that high risk women with a history of invasive cervical surgery, and those with incidental findings of a short cervix may have a different pathophysiology to those women with a history of preterm birth, sub-group analysis will be performed according to risk factor. As we are powering for equivalence in the maternal outcome, it is not anticipated that the neonatal outcomes will be different however we will collect data on composite neonatal end point (not specifically powered for equivalence).

For biomarker analysis, results will be analysed at each time point in a cross sectional analysis, and on a case–control basis. We will express the overall usefulness of each marker for prediction of the primary outcome as a ROC area (receiver operating characteristic), with 95% confidence interval and *p*-value. We will describe the performance of the most useful markers in terms of sensitivity, specificity and related measures for selected cut-points. We will use logistic regression in order to identify possible useful combinations of markers; where possible, we will use the repeated measurements to describe the change in test performance with gestation.

### Side effects and adverse events reporting

Study participants are advised to contact the chief investigator or research team at any time if symptoms develop. Expected SAEs are those events which are expected in the patient population or as a result of the routine care/treatment of a patient. The trial interventions are those which would be routinely offered in clinical practice. Cervical cerclage insertion is an established surgical procedure, which is associated with minimal risks. These include infection, miscarriage, bleeding, rupture of membranes and difficulty with suture removal. The cervical pessary is not associated with known risk. There is a risk of allergy with vaginal progesterone. Symptoms of overdose may include somnolence, dizziness, euphoria or dysmenorrhoea (latter not applicable for pregnant women). Treatment is largely observation with symptomatic and supportive measures as necessary.

Serious adverse events/reactions which are unrelated to these clinical procedures will be reported as SAEs.

Events that are primary or secondary outcome measures are not considered to be SAEs and will be reported in the normal way, on the appropriate electronic case report form. These include:

Maternal: Premature labour, premature rupture of membranes, chorioamnionitis

Infant: Perinatal death (unless unexpected in this population), low birth weight, requirement for supplemental oxygen or ventilation support, complications of prematurity (e.g. intraventricular haemorrhage, necrotising enterocolitis, encephalopathy, seizures, hypoglycaemia) unless unexpected in this population

In addition, the following common pregnancy complication events will not be considered SAEs: hospitalisation for pre-eclampsia or pregnancy induced hypertension, hospitalisation for symptoms of preterm labour (e.g. rupture of membranes, vaginal bleeding); hospitalisation for maternal discomfort; hospitalisation for rest; hospitalisation for observation or monitoring for a period of less than 12 hrs; delivery complications such as caesarean section of postpartum haemorrhage.

### Interim analysis and treatment stopping rules

There is no planned interim analysis. The trial may be prematurely discontinued by the Sponsor, Chief Investigator or Regulatory Authority on the basis of new safety information or for other reasons given by the DMEC/TSC regulatory authority or ethics committee concerned. If the trial is prematurely discontinued, active participants will be informed and no further participant data will be collected.

### Protocol guidelines

All SPIRIT guidelines were adhered to in the creation of this protocol.

### Committee oversights

An independent Trial Steering Committee and an independent Data Monitoring Committee has been appointed to oversee trial management and safety of the participants in the trial.

## Discussion

Preterm birth is the leading cause of perinatal morbidity and mortality and a short cervix is a useful way of identifying those most at risk. However, best management of these women has presented a clinical conundrum for decades.

Given the promise offered by cerclage, Arabin pessary and vaginal progesterone for prevention of preterm birth in individual trials, direct comparison of these prophylactic interventions is now essential to establish whether one treatment is superior. If, as we hypothesise, the three interventions are equally efficacious, this study will empower women to make a choice of treatments based on personal preference and quality of life issues also explored by the study.

We know that the aetiologies of preterm birth are diverse. Whilst pragmatic, the study of this heterogenous group together may be artificial and responsible for conflicting results from previous studies. Our exploratory analysis into whether the response to intervention is related to the pre-intervention biomarker status may help us identify subgroups of women who respond differently to each treatment. This will enable more targeted treatments for high risk women in the future. It will also further our understanding of the pathophysiology of spontaneous preterm birth and help focus future research questions.

## Abbreviations

CI: Confidence interval
CVF: Cervicovaginal fluid
NIHR: National Institute for Health Research
NNT: Number needed to treat
RCOG: Royal College of Obstetrics & Gynaecology
ROC: Receiver operator curve
RR: Relative risk
SAE: Serious adverse event
sPTB: Spontaneous preterm birth
TVS: Transvaginal scan
